# Air Cure

**Published:** 1859-08

**Authors:** 


### AIR CURE.

Nature every where abounds with remedial agents, but it often requires years, ages, centuries, for us to ascertain their aptitude, and the best mode of application.

There are some persons who have so little life in them, so little recuperative power, that it requires a long time for the scratch of a pin to heal. Others have sores, which become so sluggish, that they never heal over, but slowly increase, to the end of life; this is frequently the case with the "sore leg" of old people.

A French surgeon has been making experiments upon sores and wounds of persons of frail constitutions, and when every form of remedies failed, internal and external, the most encouraging success has been found in causing a common hand bellows to act on the part for fifteen minutes at a time, four times a day. The immediate effect was a feeling of refreshing coolness at the spot; pain was moderated; and the surface became paler, less angry, and by the next day, a crust began to form, ending in a cure. As there are cases where persons cannot bear internal medicines, while external applications are not unattended with danger, it may be well enough to know a mild, safe, efficient remedy always at hand, costing nothing.

